# Repurposing Coronary Risk Scores to Identify Increased Likelihood of Atrial Fibrillation in Chronic Coronary Syndrome

**DOI:** 10.3390/medsci14020161

**Published:** 2026-03-24

**Authors:** Alexandru-Florinel Oancea, Mathilde Leonard, Paula Cristina Morariu, Maria Godun, Alexandru Jigoranu, Ionela-Larisa Miftode, Radu Stefan Miftode, Aurelia Mihaela Nica, Alexandra Rotaru, Paul Simion, Ana Maria Buburuz, Diana-Elena Floria, Raluca Mitea, Cristina Gena Dascalu, Elena Cojocaru, Antoniu Octavian Petriș, Irina-Iuliana Costache-Enache, Mariana Floria

**Affiliations:** 1 Grigore T. Popa University of Medicine and Pharmacy Iasi, 700115 Iasi, Romania; alexandru.oancea@umfiasi.ro (A.-F.O.); mathilde.leonard@yahoo.com (M.L.); morariu.paula-cristina@email.umfiasi.ro (P.C.M.); godun.maria-mihaela@d.umfiasi.ro (M.G.); alexandru.jigoranu@umfiasi.ro (A.J.); ionela-larisa.miftode@umfiasi.ro (I.-L.M.); radu-stefan.miftode@umfiasi.ro (R.S.M.); nica_aurelia-mihaela@d.umfiasi.ro (A.M.N.); alexandra-manuela.rotaru@umfiasi.ro (A.R.); paul-alexandru.simion@d.umfiasi.ro (P.S.); diana-elena.iov@d.umfiasi.ro (D.-E.F.); cdascalu_info@yahoo.com (C.G.D.); elena2.cojocaru@umfiasi.ro (E.C.); antoniu.petris@umfiasi.ro (A.O.P.); irina.costache@umfiasi.ro (I.-I.C.-E.); floria.mariana@umfiasi.ro (M.F.); 2 Saint Spiridon Emergency Hospital, 700115 Iasi, Romania; 3 St Parascheva Clinical Hospital of Infectious Diseases, 700116 Iasi, Romania; 4 Faculty of Medicine Victor Papilian, University of Lucian Blaga, 550169 Sibiu, Romania; daria.mitea@ulbsibiu.ro; 5 Department of Morphofunctional Sciences—Pathology, Pediatric Hospital, 700115 Iasi, Romania

**Keywords:** atrial fibrillation, chronic coronary syndrome, clinical risk scores, SYNTAX score, Gensini score, cardiovascular risk stratification, coronary artery disease

## Abstract

Atrial fibrillation (AF) frequently coexists with chronic coronary syndrome (CCS), reflecting shared cardiovascular risk factors and structural remodeling pathways. Identifying CCS patients at increased likelihood of AF remains clinically relevant, particularly when arrhythmia is silent or paroxysmal. **Background:** We hypothesized that established clinical and angiographic risk scores used in CCS may capture cumulative cardiovascular burden and could therefore assist in AF risk stratification. The biomarker-based ABC-stroke score was incorporated as a biological reference framework reflecting myocardial stress and injury. **Methods**: This prospective, single-center proof-of-concept study included 131 consecutive patients undergoing invasive coronary angiography for suspected myocardial ischemia. Patients were classified according to rhythm status, irrespective of AF subtype. Coronary artery disease severity was quantified using the Gensini and SYNTAX (PCI and CABG) scores. Global cardiovascular risk was assessed using Framingham, ASCVD, SCORE2, and SCORE2-OP. Correlation analyses, ROC curves, and multivariable logistic regression were performed to evaluate associations between risk scores, coronary complexity, and AF. **Results**: Clinical and angiographic risk scores differed significantly according to rhythm status and AF phenotype. Patients with AF exhibited higher global cardiovascular risk and greater coronary anatomical complexity compared with those in sinus rhythm. SYNTAX PCI and SYNTAX CABG demonstrated moderate discriminative performance for AF detection (AUC 0.745 and 0.760, respectively), with SYNTAX CABG remaining independently associated with AF in multivariable analysis. Significant correlations were observed between traditional cardiovascular risk scores and SYNTAX-derived measures of coronary complexity, whereas correlations with the Gensini score were weaker. The ABC-stroke reference model showed a strong discriminative signal, consistent with its biological proximity to AF-related myocardial stress. **Conclusions**: Established clinical and angiographic risk scores used in CCS are associated with the presence and phenotype of AF. These findings suggest that routinely available coronary risk assessment tools may serve as practical instruments for identifying CCS patients at increased likelihood of AF, potentially facilitating targeted rhythm screening and earlier risk stratification.

## 1. Introduction

Given the marked clinical and prognostic heterogeneity of patients with chronic coronary syndrome (CCS), accurate risk stratification is essential for guiding diagnostic evaluation, therapeutic decision-making, and long-term management. A wide range of risk scores have been developed in this setting and can be broadly categorized into anatomical scores, derived from coronary angiographic complexity, and clinical scores, designed to estimate global cardiovascular risk. Beyond their established role in coronary disease assessment, these tools capture cumulative exposure to cardiovascular risk factors and the burden of structural heart disease—pathophysiological substrates that also contribute to the development and maintenance of atrial fibrillation (AF). In this regard, the biomarker-based ABC risk framework offers a more individualized estimation of cardiovascular vulnerability in patients with AF by integrating age, prior cerebrovascular events, and circulating markers of myocardial injury and wall stress. Unlike traditional clinical scores based exclusively on historical risk factors, the ABC model reflects ongoing myocardial injury and hemodynamic stress—biological processes that are likewise prevalent in CCS due to chronic ischemia and diffuse atherosclerotic involvement. Therefore, elevated ABC values in patients with coexisting AF and CCS should be interpreted not as direct indicators of arrhythmia, but as markers of shared systemic cardiovascular disease severity, associated with heightened risks of stroke, bleeding, and mortality. This score was incorporated in this study not as a tool for AF detection, but as a biological reference framework reflecting myocardial injury and hemodynamic stress. In this context, the ABC score was used to explore whether biomarker-defined cardiovascular vulnerability aligns with coronary anatomical complexity and global cardiovascular risk burden in patients with CCS [1,2,3].

Several studies have explored the relationship between traditional cardiovascular risk scores and the development of AF. Risk models derived from population-based cohorts, including the Framingham AF risk score and other multimorbidity-based frameworks, have demonstrated that cumulative cardiometabolic burden contributes substantially to arrhythmia development. Similarly, observational studies have reported associations between coronary artery disease (CAD) complexity and AF prevalence, particularly in patients undergoing coronary revascularization. For example, a Bayesian network (BN) model using factors like age, gender, systolic blood pressure, LDL-C, uric acid, NYHA class, diabetes, hypertension, symptoms, and left atrial diameter achieved an AUC (area under curve) of 0.90 on test sets and 0.89 on cross-validation, outperforming some traditional algorithms for large-scale screening. Machine learning approaches, including those from the AFIRE trial, integrate clinical and lab data to predict adverse events in AF with stable CAD, showing superior discrimination over conventional scores [4].

Moreover, multimorbidity-based AF prediction frameworks integrate multiple chronic conditions—such as hypertension, diabetes, heart failure, chronic kidney disease, and CAD—into risk models to better forecast AF onset and progression. These approaches, often employing machine learning or Bayesian networks, account for synergistic interactions among comorbidities, revealing how clusters like cardiometabolic multimorbidity elevate AF risk up to 6-fold while complicating management in CAD patients. For instance, studies from cohorts like Lifelines and RACE II demonstrate that higher multimorbidity counts independently predict worse cardiovascular outcomes, enabling tailored interventions like early screening or polypharmacy optimization [5].

However, few investigations have evaluated whether risk scores routinely applied for coronary disease stratification—both clinical and angiographic—may collectively signal an increased likelihood of AF in patients with CCS. The present study therefore aims to explore this integrative framework.

### 1.1. Anatomical Scores for the Assessment of Coronary Artery Disease Severity

The Gensini score is a well-established angiographic tool for quantifying the severity of CAD. It assigns weighted values to coronary stenoses according to the degree of luminal narrowing and the functional significance of the affected segment. Greater weighting is attributed to lesions involving the left main coronary artery and proximal segments, reflecting their substantial hemodynamic relevance. By integrating both lesion severity and anatomical distribution, the Gensini score provides a comprehensive estimate of total atherosclerotic burden. It has been widely used in studies examining the relationship between anatomical disease extent, inflammatory activation, and long-term cardiovascular prognosis. In patients with CCS, an increased atherosclerotic burden may also promote atrial structural remodeling through chronic ischemia, microvascular dysfunction, and myocardial fibrosis, thereby creating a substrate conducive to AF [6,7,8].

The SYNTAX score was developed to characterize the anatomical complexity of CAD beyond stenosis severity alone. It incorporates detailed lesion-specific features, including location, bifurcation or trifurcation involvement, chronic total occlusions, severe calcification, vessel tortuosity, thrombus burden, and diffuse disease extension. As such, SYNTAX reflects overall coronary lesion complexity and plays a central role in guiding revascularization strategies, particularly when choosing between percutaneous coronary intervention (PCI) and coronary artery bypass grafting (CABG) in patients with multivessel or left main disease. In the setting of CCS, higher SYNTAX scores are associated with greater procedural complexity, increased risk of adverse cardiovascular events, and less favorable long-term outcomes. Importantly, diffuse and complex coronary disease quantified by SYNTAX may parallel systemic vascular remodeling and atrial structural changes, thereby contributing to the development and maintenance of AF [9,10,11].

Moreover, higher SYNTAX scores correlate with increased risk of atrial arrhythmias, particularly postoperative AF (PoAF) after CABG. In one study of 94 CABG patients, SYNTAX score was an independent predictor of PoAF (OR 1.139 per unit increase), with scores > 18.75 offering 85.7% sensitivity; PoAF occurred in 33.3% overall, more frequently in high-score groups alongside age and chronic obstructive pulmonary disease (COPD). Multimorbidity-based AF prediction frameworks emphasize clustered comorbidities like hypertension, diabetes, heart failure, and CAD, which amplify AF incidence and outcomes; models reveal complex interactions raising heart failure risk 6-fold in AF cases. These approaches support integrated care to mitigate risks in multimorbid CAD-AF patients [12].

### 1.2. Clinical Scores for Global Cardiovascular Risk Estimation

In addition to anatomical evaluation, assessment of global cardiovascular risk is fundamental in patients with CCS, particularly for guiding secondary prevention and long-term management. The Framingham risk score, one of the earliest and most extensively validated prediction models derived from the Framingham Heart Study, estimates 10-year coronary event risk based on age, sex, lipid profile, blood pressure, antihypertensive treatment, smoking status, and diabetes mellitus. Although originally developed for primary prevention, it has been frequently applied in CCS populations to underscore the contribution of traditional cardiovascular risk factors to atherosclerotic progression and adverse outcomes. Notably, these same risk determinants are well-established contributors to the development and persistence of AF [13,14].

The Atherosclerotic Cardiovascular Disease (ASCVD) score was designed to estimate the risk of major atherosclerotic events, including myocardial infarction and stroke. By integrating demographic characteristics and cardiometabolic risk factors, it provides a clinically oriented assessment of future cardiovascular events and is widely used to guide lipid-lowering and preventive strategies. In patients with CCS, ASCVD scoring may help identify residual cardiovascular risk and inform intensified prevention; however, given the shared pathophysiological substrate, it may also reflect susceptibility to AF [15].

In Europe, cardiovascular risk prediction has been refined through the introduction of SCORE2 and SCORE2-OP, which estimate the 10-year risk of fatal and non-fatal cardiovascular events. SCORE2 applies to individuals aged 40–69 years, whereas SCORE2-OP is tailored for those aged ≥70 years. These models incorporate age, sex, smoking status, systolic blood pressure, and non-HDL cholesterol and are calibrated to regional cardiovascular risk profiles. While primarily intended for primary prevention, SCORE2 models remain relevant in CCS—particularly in patients with early-stage disease or multiple risk factors—by providing a standardized estimate of global atherosclerotic burden [16].

**Figure 1:**
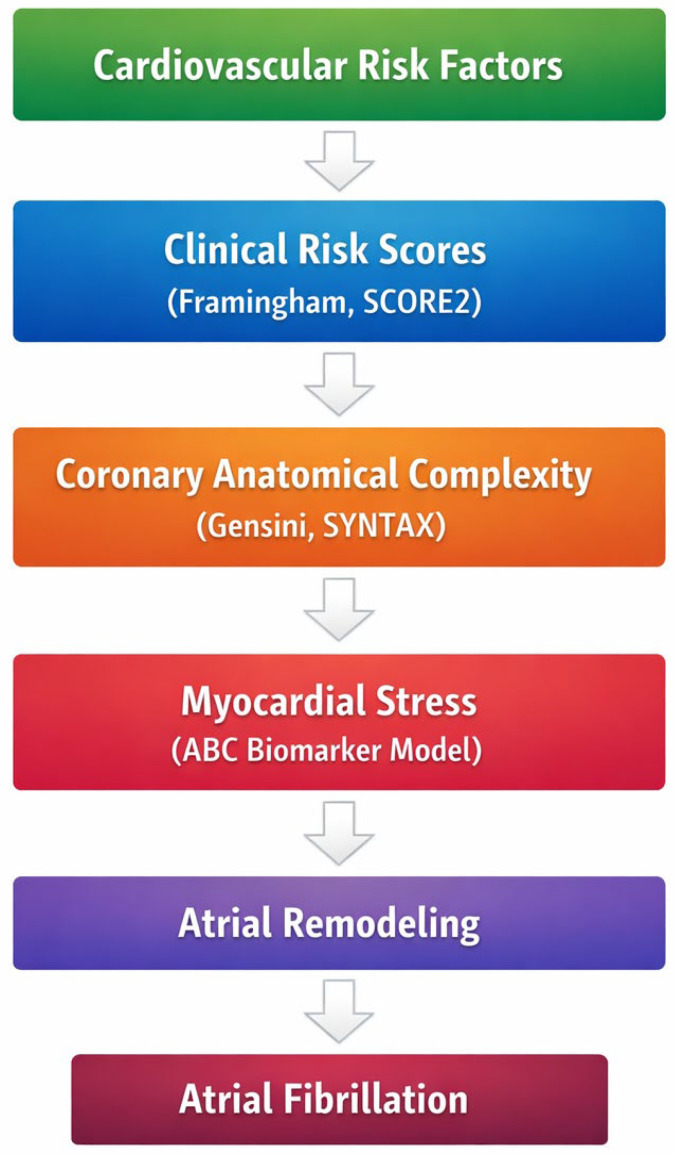
Conceptual framework linking cardiovascular risk burden, coronary anatomical complexity, biomarker-defined myocardial stress, and AF in CCS.

To illustrate the conceptual framework underlying the present study, we propose an integrative model linking cumulative cardiovascular risk burden, coronary anatomical complexity, and biomarker-defined myocardial stress with the development of AF in patients with CCS. Within this framework, traditional cardiovascular risk scores reflect long-term cardiometabolic exposure, coronary anatomical scores capture structural disease complexity, and biomarker-based models provide a biological layer reflecting myocardial stress and injury. The convergence of these factors may contribute to atrial structural remodeling and increased susceptibility to AF (Figure 1).

An important challenge in patients with coexisting AF and CAD concerns antithrombotic therapy management, particularly in individuals undergoing PCI. In this setting, clinicians must balance the competing risks of thromboembolism, stent thrombosis, and bleeding while combining oral anticoagulant and antiplatelet therapies. A recent study reviews evidence showing that while triple therapy (OAC plus DAPT with aspirin and P2Y12 inhibitor) was traditionally used initially post-PCI, dual therapy (OAC + P2Y12 inhibitor like clopidogrel) reduces major and intracranial bleeding without significantly increasing stent thrombosis, as demonstrated in trials like WOEST, PIONEER AF-PCI, RE-DUAL PCI, AUGUSTUS, and ENTRUST-AF PCI. Guidelines now recommend short triple therapy (1–30 days, often aspirin discontinuation within 7 days or at discharge), transitioning to dual therapy up to 1 year, then OAC monotherapy lifelong, preferring DOACs over VKAs and P2Y12 inhibitors over aspirin in dual regimens; beyond one year; AFIRE supports OAC monotherapy superiority. Future trials like MATRIX-2 and OPTIMA-AF explore even shorter dual therapy or aspirin-free strategies from PCI onset [17].

Accordingly, the objective of this prospective observational cross-sectional proof-of-concept study was to determine whether established clinical and angiographic risk scores routinely applied in CCS are associated with the presence and phenotype of AF in patients undergoing invasive coronary assessment. In addition, we incorporated the biomarker-based ABC-stroke score as a biological reference framework rather than as a competing model. By reflecting active myocardial injury and hemodynamic stress, the ABC score enabled us to explore whether coronary anatomical complexity and global cardiovascular risk burden converge with a biomarker-defined layer of cardiovascular vulnerability. Through this integrative approach, we sought to examine whether AF in CCS represents a manifestation of systemic cardiovascular disease continuum rather than an isolated electrical disorder.

## 2. Materials and Methods

### 2.1. Study Design and Patient Population

This prospective, observational, cross-sectional, single-center, proof-of-concept study was conducted at the Emergency Clinical Hospital Saint Spiridon from Iași, Romania. Consecutive patients admitted with an indication for invasive coronary angiography for suspected CCS were enrolled between January and May 2024.

The study population consisted of 131 consecutive patients aged ≥18 years, with or without a diagnosis of AF, regardless of AF subtype (paroxysmal, persistent, or permanent). AF was identified based on previously documented AF in the medical history or detected during the index hospitalization through electrocardiographic evaluation. Rhythm assessment included standard 12-lead electrocardiography and 24 h Holter ECG monitoring for all patients. Consequently, the AF group included both patients with known AF and those with newly documented AF episodes during hospitalization.

All patients were evaluated for suspected stable CAD based on clinical presentation and/or non-invasive testing. Eligible patients met one or more of the following inclusion criteria: presence of major cardiovascular risk factors, including arterial hypertension, dyslipidemia, diabetes mellitus, obesity, active smoking, or a strong family history of coronary artery disease; typical angina symptoms; positive non-invasive ischemia testing (exercise stress test, stress echocardiography, or myocardial perfusion scintigraphy) or evidence of coronary artery stenosis on coronary computed tomography angiography (CCTA) with an indication for invasive confirmation. All participants were required to have the capacity to understand the study protocol and to provide written informed consent prior to enrollment.

Although the same patient cohort has been previously used in published analyses evaluating inflammatory biomarkers [18] and cardiovascular risk scores [19] in the context of CCS, the present study addresses a distinct research objective. Specifically, the current analysis focuses on the association between clinical risk scores used in CCS and the presence of AF. No biomarker-based analyses are included in the present study.

### 2.2. Exclusion Criteria

Patients were excluded if they met any of the following criteria:

- Acute coronary syndrome, including ST-segment elevation myocardial infarction (STEMI), non–ST-segment elevation myocardial infarction (NSTEMI), or unstable angina.
- Advanced heart failure (NYHA functional class IV).
- Cardiomyopathies of any etiology (dilated, hypertrophic, restrictive, or arrhythmogenic).
- Hemodynamically significant valvular heart disease (greater than moderate severity).
- Presence of a permanent cardiac pacing device.
- Advanced renal or hepatic disease.
- Active infection, systemic inflammatory disease, or malignancy.
- Uncontrolled thyroid disorders or psychiatric conditions affecting compliance or biological status.
- Inability to provide informed consent (e.g., unconscious or intubated patients).
- Explicit refusal to participate in the study.

### 2.3. Clinical and Paraclinical Data Collection

After verification of eligibility criteria, a prospective database was created for all included patients. Data collection was performed in a standardized manner at the time of inclusion, integrating information obtained from medical history, physical examination, laboratory investigations, imaging studies, and the hospital electronic medical record system. Collected demographic and clinical variables included age, sex, smoking status, and cardiovascular comorbidities such as arterial hypertension, diabetes mellitus, heart failure, chronic kidney disease (moderate impairment), chronic obstructive pulmonary disease, dyslipidemia, peripheral arterial disease, transient ischemic attack, stroke, and documented AF. Baseline chronic medications were also recorded, including statins, beta-blockers, angiotensin-converting enzyme inhibitors or angiotensin receptor blockers, oral anticoagulants, antiplatelet agents, and proton pump inhibitors.

### 2.4. Coronary Angiography and Anatomical Scoring

Invasive coronary angiography was performed using a Philips Azurion 7 (Philips Healthcare, Best, the Netherlands) system. Coronary lesion severity was primarily assessed by visual estimation by experienced interventional cardiologists. Fractional flow reserve (FFR) or instantaneous wave-free ratio (iFR) measurements were selectively employed for angiographically borderline lesions, at the operator’s discretion.

The main risk scores used in CCS, together with their typology and calculation principles, are summarized in Table 1, highlighting the complementary nature of coronary anatomical assessment and global cardiovascular risk estimation. The biomarker-based ABC-stroke risk score was calculated using the validated web-based calculator provided by the Uppsala Clinical Research Center. This model integrates age, prior stroke or transient ischemic attack, high-sensitivity cardiac troponin, and NT-proBNP, variables identified as key predictors of thromboembolic risk in AF. The calculator was used to ensure standardized and reproducible estimation of ABC-stroke risk across the study population.

The anatomical severity and complexity of coronary artery disease were quantified using validated angiographic scoring systems according to the predefined study protocol. The Gensini score was calculated to estimate global coronary atherosclerotic burden, while the SYNTAX score (PCI and CABG variants) was used to assess coronary anatomical complexity.

Global cardiovascular risk was evaluated using established clinical prediction models, including the Framingham, ASCVD, SCORE2, and SCORE2-OP scores, calculated according to their original definitions (Table 1). In addition, the biomarker-based ABC-stroke score was determined for all patients. The ABC-stroke model integrates age, prior stroke or transient ischemic attack, high-sensitivity cardiac troponin, and N-terminal pro–B-type natriuretic peptide (NT-proBNP) to estimate thromboembolic risk in patients with AF.

All clinical scores were calculated independently by two trained investigators blinded to angiographic findings and rhythm status. Discrepancies were resolved by joint reassessment and consensus. These scores were subsequently analyzed in relation to rhythm status and AF subtype using multivariable logistic regression and ROC curve analysis to evaluate their association with AF in patients with CCS.

### 2.5. Statistical Analysis

Statistical analyses were performed using IBM SPSS Statistics, version 29.0 (IBM Corp., Armonk, NY, USA). Continuous variables were summarized using descriptive statistics, including mean ± standard deviation, median, and minimum–maximum values.

Normality of data distribution was assessed using the Kolmogorov–Smirnov test. Depending on data distribution, comparisons between two groups were performed using Student’s *t*-test or the Mann–Whitney U test, while comparisons across multiple groups were conducted using one-way ANOVA or the Kruskal–Wallis test, as appropriate. Post hoc analyses were applied when global tests reached statistical significance. Associations between continuous variables were evaluated using Spearman’s rank correlation coefficient (rho), with corresponding *p*-values and 95% confidence intervals, to assess the strength and direction of correlations.

A two-sided *p*-value < 0.05 was considered statistically significant, while values <0.01 were considered highly statistically significant. To evaluate the discriminative ability of clinical and angiographic scores for identifying AF, receiver operating characteristic (ROC) curve analysis was performed. The AUC, 95% confidence intervals, optimal cut-off values, sensitivity, and specificity were calculated for each parameter.

For multivariate analysis, binary logistic regression was conducted using the Forward likelihood ratio (LR) method. Variables included in the initial model were those that demonstrated statistically significant differences between patients with and without AF in univariate analyses. Model calibration and performance were assessed using the Hosmer–Lemeshow goodness-of-fit test, Nagelkerke’s R^2^, overall classification accuracy, sensitivity, and specificity.

Potential multicollinearity among variables was evaluated using variance inflation factors (VIFs), with all values <2.5, indicating the absence of significant collinearity.

### 2.6. Ethics

This proof-of-concept study was conducted in accordance with the ethical principles outlined in the Declaration of Helsinki, as revised in 2013. Upon admission, all participants provided written informed consent after receiving detailed explanations regarding the study objectives, procedures, and their rights as participants. The study protocol was reviewed and approved by the Ethics Committee of the University of Medicine and Pharmacy “Gr. T. Popa” Iași (Approval No. 352; date of approval: 9 October 2023) and the Ethics Committee of St. Spiridon Emergency Clinical Hospital, Iași (Approval No. 75; date of approval: 11 September 2023).

## 3. Results

Among the 131 patients included in the study, 65 patients were diagnosed with significant CCS (S-CCS), defined by the presence of ≥70% stenosis in at least one coronary artery with a diameter ≥ 2 mm or ≥50% stenosis of the left main coronary artery. The remaining 66 patients were classified as non-significant CCS (N-SCC), defined by coronary stenoses below these thresholds. This latter group also included patients with angina with non-obstructive coronary arteries (ANOCA), an entity increasingly recognized in recent literature, characterized by distinct pathophysiological mechanisms and growing clinical relevance [20].

Regarding cardiac rhythm, 54.2% of the study population had AF. Among these patients, 26.7% had permanent AF, 16.8% had paroxysmal AF, and 10.7% had persistent AF, while the remaining patients were in sinus rhythm (SR).

Subsequently, based on coronary disease severity and rhythm status, the study population was stratified into four patient groups, as follows (Table 2):

- 25 patients with SR and N-CCS;
- 35 patients with SR and S-CCS;
- 41 patients with AF and N-CCS;
- 30 patients with AF and S-CCS.

It can be observed that the proportion of patients in SR was higher among those with S-SCC compared with patients with non-significant lesions (53.8% vs. 37.9%). Among patients with permanent AF, a higher proportion was noted in the N-SCC group (36.4% vs. 16.9%). A similar pattern was observed for persistent AF, which occurred at nearly double the frequency in patients with non-significant lesions compared with those with significant CCS (13.6% vs. 7.7%). In contrast, among patients with significant coronary lesions, paroxysmal AF was more prevalent, being observed in 21.5% of cases compared with 12.1% in the non-significant CCS group. These distributions are detailed in Table 2.

The results presented in this section focus exclusively on the analysis of clinical and angiographic risk scores in relation to AF in patients with CCS. Other clinical, biological, echocardiographic, and treatment-related characteristics of the study population have been previously reported and discussed in detail in earlier publications derived from the same cohort, as mentioned before. Therefore, these variables are not reiterated in the present manuscript. The current analysis specifically addresses the primary objective of this study, namely the evaluation of established risk scores used in CCS as potential risk enrichment for AF detection and subtype.

### 3.1. Potential Assessment of AF Using CCS-Related Risk Scores

To evaluate the relationship between AF subtype and coronary artery disease severity, several validated clinical risk scores, including cardiovascular risk scores and angiographic indices, were compared between patients in SR and those with different AF phenotypes. The biomarker-based ABC-stroke score was included in this comparative framework as a biological reference layer reflecting active myocardial stress, rather than as a competing construct. The results are summarized in Table 3, which presents the distribution of each parameter across the analyzed groups. Non-parametric tests (Mann–Whitney and Kruskal–Wallis) were applied, or ANOVA when test assumptions were met. The table reports both mean and median values for each subgroup, together with global and post hoc statistical significance, highlighting relevant differences among AF subtypes.

Consistent with the predefined biological reference framework, the ABC-stroke score demonstrated a clear and progressive increase across rhythm categories. Mean values rose from 0.45 ± 0.31 in patients with SR to 0.86 ± 0.53 in paroxysmal AF, 0.90 ± 0.44 in persistent AF, and 1.13 ± 0.63 in permanent AF. This ascending pattern was reflected in median values and distribution extremes, and overall group differences were highly statistically significant (*p* < 0.001). Post hoc analyses confirmed that patients in SR had significantly lower ABC-stroke values compared with each AF subtype, supporting a graded increase in biomarker-defined cardiovascular vulnerability across AF phenotypes.

Global cardiovascular risk scores exhibited a similar directional pattern. The ASCVD score reached its highest mean value in paroxysmal AF (35.55 ± 14.10), followed by persistent (21.62 ± 9.77) and permanent AF (20.92 ± 12.45), whereas SR patients showed the lowest levels (19.54 ± 13.23) (*p* < 0.001). The Framingham score followed a comparable gradient, with minimal values in SR (11.30 ± 7.60), peak levels in paroxysmal AF (18.65 ± 6.99), and intermediate values in persistent and permanent AF (*p* = 0.003). SCORE2-OP values also differed significantly among groups (*p* = 0.004), with the highest levels observed in paroxysmal AF (27.86 ± 8.88) and the lowest in SR (18.89 ± 10.42). Pairwise comparisons consistently highlighted significant differences between SR and paroxysmal AF.

Anatomical complexity assessed by SYNTAX scoring demonstrated a progressive increase from SR toward more sustained AF forms. The SYNTAX PCI score rose from 29.50 ± 8.82 in SR to 35.90 ± 8.62 in paroxysmal AF, reaching the highest values in persistent AF (43.44 ± 13.28), with slightly lower but still elevated values in permanent AF (38.81 ± 12.97) (*p* = 0.007). A similar ascending pattern was observed for SYNTAX CABG, with mean values of 26.35 ± 9.04 in SR, 34.90 ± 9.41 in paroxysmal AF, and 41.04 ± 11.44 in persistent AF (*p* = 0.001). Post hoc analyses demonstrated significant contrasts between SR and AF subtypes, particularly paroxysmal and persistent AF.

In contrast, the Gensini score displayed a decreasing trend across rhythm categories, with higher values in SR (30.24 ± 38.64) and paroxysmal AF (30.86 ± 39.82), lower values in persistent AF (25.00 ± 32.68), and the lowest levels in permanent AF (15.29 ± 22.14) (*p* = 0.043). This inverse pattern suggests that overall stenotic burden may not parallel anatomical lesion complexity in relation to AF phenotype.

Taken together, these findings demonstrate structured and graded differences across rhythm categories, with biomarker-defined vulnerability, global clinical risk, and coronary anatomical complexity generally increasing from SR toward more sustained AF forms. This convergence supports the concept that AF phenotype in CCS reflects cumulative cardiovascular burden rather than an isolated electrical disturbance.

### 3.2. Diagnostic Performance of CCS-Related Risk Scores in AF

Subsequently, the seven parameters that demonstrated statistically significant differences between patients with and without AF were entered into a binary logistic regression model using the Forward LR method. The Hosmer–Lemeshow goodness-of-fit test indicated adequate model performance, as the result was not statistically significant (*p* = 0.471). The initial discriminative performance for AF was 53.8%, increasing to 86.2% in the final model, indicating good discriminative ability of the identified variables. The model explained 50.8% of the variance in AF diagnosis (Nagelkerke R^2^ = 0.508) and demonstrated a sensitivity of 80.0% and a specificity of 91.4%.

Among the seven tested variables, both the ABC score and SYNTAX CABG remained statistically significant. As expected for a biomarker-based model reflecting myocardial stress and injury, the ABC score showed a strong association with AF (OR 35.160, *p* = 0.002). Importantly, SYNTAX CABG also retained independent association (OR 1.080, *p* = 0.040), suggesting that coronary anatomical complexity contributes to AF presence beyond biomarker-related proximity. A one-unit increase in the ABC score increased the odds of AF by 35.160-fold, while a one-unit increase in SYNTAX CABG increased the odds of AF by 1.080-fold, with all other variables held constant (Table 4).

The ABC score showed a high discriminative signal (AUC 0.908; sensitivity 83.1%; specificity 88.3% at a cut-off value of 0.615), consistent with its biomarker-based derivation within AF populations and its reflection of active myocardial stress. In contrast, coronary anatomical scores demonstrated moderate but clinically meaningful discriminative performance. SYNTAX PCI exhibited an AUC of 0.745 (sensitivity 83.3%; specificity 62.9% at a cut-off of 28.95), while SYNTAX CABG showed an AUC of 0.760 (sensitivity 70.0%; specificity 74.3% at a cut-off of 29.95). These findings suggest that structural coronary complexity aligns with AF status within a layered cardiovascular framework, rather than representing a direct competition with biomarker-based models.

**Figure 2:**
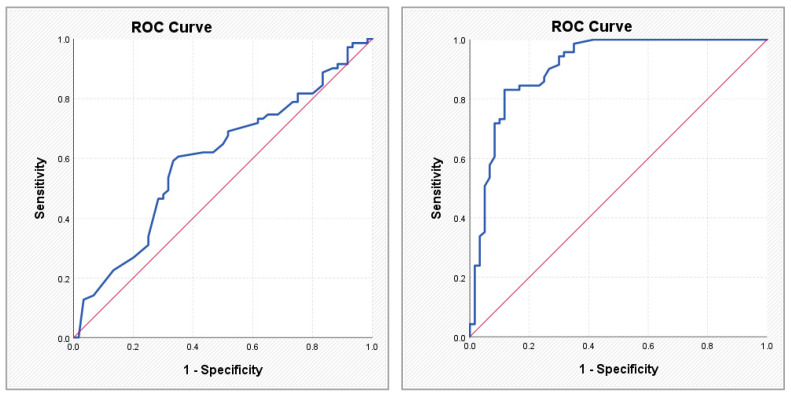
ROC curves illustrating the performance of the Gensini score (**left**) and the ABC score (**right**) in predicting atrial fibrillation.

**Figure 3:**
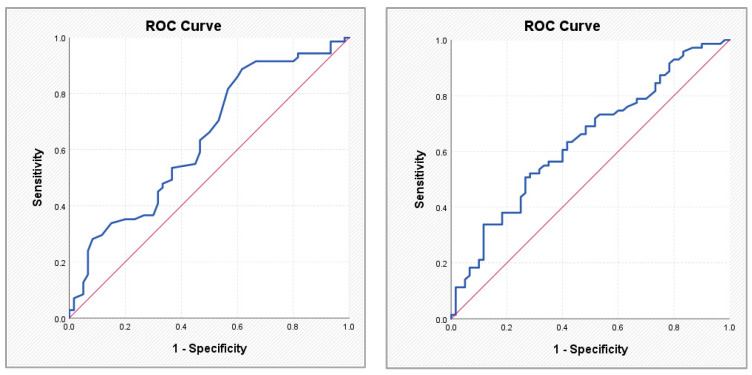
ROC curves illustrating the performance of the SCORE2-OP score (**left**) and the Framingham score (**right**) in predicting atrial fibrillation.

**Figure 4:**
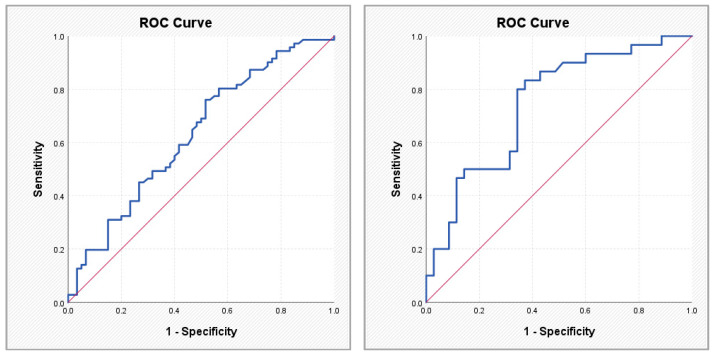
ROC curves illustrating the performance of the ASCVD score (**left**) and the SYNTAX PCI score (**right**) in predicting atrial fibrillation.

**Figure 5:**
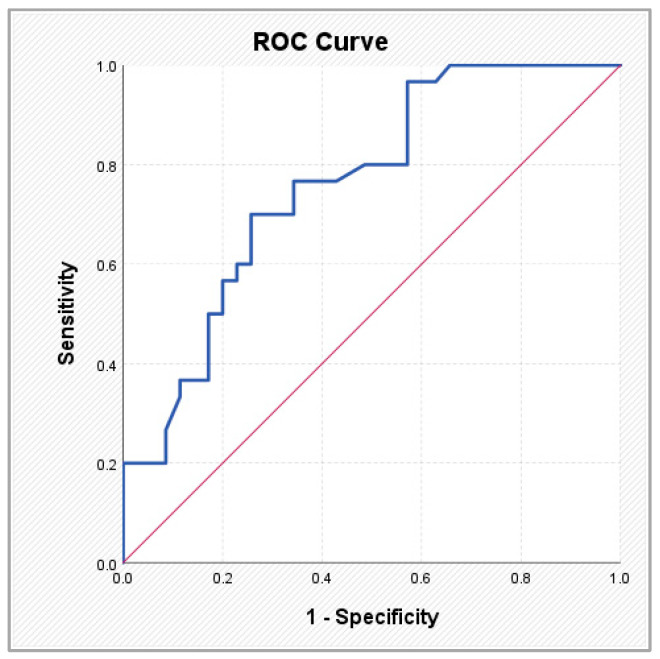
ROC curve illustrating the performance of the SYNTAX CABG score in predicting atrial fibrillation.

To assess the utility of clinical, anatomical, and atherosclerotic burden scores in identifying patients with AF, a ROC analysis was performed for the parameters that showed statistically significant differences in comparisons between patients with and without AF. Table 5 and Figure 2, Figure 3, Figure 4 and Figure 5 present the discriminative performance of each marker, including the AUC values, 95% confidence intervals, optimal cut-off thresholds, and corresponding sensitivity and specificity. These results quantify the ability of scores traditionally used in the coronary setting—such as SYNTAX PCI/CABG and global cardiovascular risk scores—to function as clinical tools for AF identification, enabling preliminary risk stratification in routine clinical practice.

**Figure 6:**
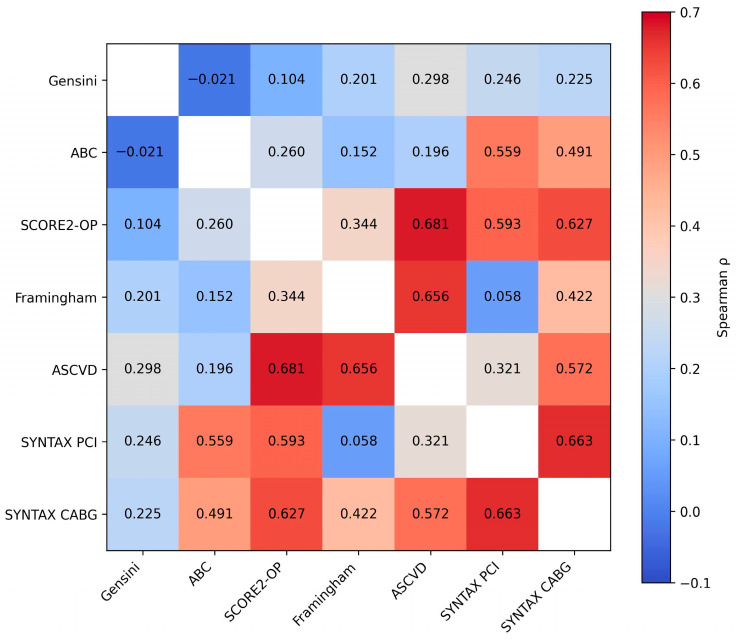
Heatmap illustrating the correlations between clinical cardiovascular risk scores, coronary anatomical complexity scores, and the biomarker-based ABC model. Color intensity reflects the strength of the Spearman correlation coefficient (*p*).

To explore the internal relationships among the different layers of cardiovascular assessment—risk factor–based scores, coronary anatomical complexity scores, and the biomarker-based ABC reference model—a Spearman correlation analysis was performed. This approach aimed to evaluate whether these distinct domains demonstrate biological convergence rather than direct equivalence. The results are presented in Table 6 and Figure 6, which includes the rho correlation coefficients, corresponding *p* values, and 95% confidence intervals for all evaluated parameter pairs.

Evaluation of the relationships between the ABC score and other parameters revealed several statistically significant correlations. As a biomarker-based reference layer reflecting myocardial stress and injury, ABC showed moderate associations with traditional risk factor–based scores such as SCORE2-OP (rho = 0.260, *p* = 0.003) and ASCVD (rho = 0.196, *p* = 0.025), while its association with the Framingham score was marginal (*p* = 0.083), suggesting only partial overlap with cumulative risk exposure.

Notably, the strongest correlations were observed between ABC and the SYNTAX scores, with moderate-to-strong associations for both SYNTAX PCI (rho = 0.559, *p* < 0.001) and SYNTAX CABG (rho = 0.491, *p* < 0.001). This pattern indicates that the biomarker-derived injury layer aligns more closely with structural coronary complexity than with traditional risk factor–based estimates. In all cases, the 95% confidence intervals did not include zero, supporting the robustness of these associations.

Regarding the relationship with the Gensini score, most correlations were weak and not statistically significant. No relevant associations were identified with the ABC score (rho = −0.021, *p* = 0.810) or SCORE2-OP (rho = 0.104, *p* = 0.237). However, a weak but statistically significant positive correlation was observed between the Gensini score and both the Framingham score (rho = 0.201, *p* = 0.021) and the ASCVD score (rho = 0.298, *p* < 0.001), suggesting partial convergence between global atherosclerotic burden and clinically estimated cardiovascular risk. The correlation with SYNTAX PCI was modest and at the threshold of statistical significance (rho = 0.246, *p* = 0.049), whereas the association with SYNTAX CABG did not reach statistical significance (rho = 0.225, *p* = 0.072).

Correlations involving SCORE2-OP were consistently statistically significant. Moderate associations were identified with Framingham (rho = 0.344, *p* < 0.001) and ASCVD (rho = 0.681, *p* < 0.001), confirming the expected relationship between scores derived from similar cardiovascular risk factors. SCORE2-OP also demonstrated moderate correlations with anatomical parameters, including SYNTAX PCI (rho = 0.593, *p* < 0.001) and SYNTAX CABG (rho = 0.627, *p* < 0.001).

Similarly, the Framingham score showed strong correlations with ASCVD (rho = 0.656, *p* < 0.001) and a significant association with SYNTAX CABG (rho = 0.422, *p* < 0.001). In contrast, the relationship with SYNTAX PCI was not statistically significant (rho = 0.058, *p* = 0.648), indicating variable associations between global cardiovascular risk and coronary lesion complexity.

The ASCVD score demonstrated significant correlations with the Gensini score (rho = 0.298, *p* < 0.001), as well as with SCORE2-OP and Framingham (rho = 0.656, *p* < 0.001), and also with the SYNTAX scores. The correlation with SYNTAX PCI was moderate (rho = 0.321, *p* = 0.009), while a stronger association was observed with SYNTAX CABG (rho = 0.572, *p* < 0.001).

Finally, the correlation between the two SYNTAX scores—PCI and CABG—was the strongest observed in the analysis (rho = 0.663, *p* < 0.001), with the confidence interval indicating a consistent association (0.495–0.784). This finding was expected, as both scores quantify the same anatomical burden of coronary artery disease, albeit from different therapeutic perspectives.

Overall, the analysis revealed a coherent network of statistically significant correlations between traditional risk factor–based scores (SCORE2-OP, Framingham, ASCVD) and anatomical scores (SYNTAX PCI/CABG), supporting the alignment between cumulative cardiovascular risk exposure and structural coronary complexity. Within this layered framework, the biomarker-based ABC reference model demonstrated closer association with anatomical complexity than with risk factor–based estimates, reinforcing the concept of structural–biological convergence in AF. In contrast, the Gensini score exhibited the weakest correlations and the fewest statistically significant associations, suggesting that focal stenosis severity may capture a more limited dimension of cardiovascular disease compared with diffuse complexity-oriented measures.

## 4. Discussion

The findings of the present study should be interpreted within the context of a cross-sectional, hypothesis-generating design, which precludes causal inference or prediction of incident AF. Specifically, the biomarker-based ABC reference model and coronary anatomical complexity scores appear to align within a shared substrate of cumulative cardiovascular injury and structural remodeling, supporting a systemic interpretation of AF in the setting of CCS. Although the strong discriminative signal of the ABC score is biologically expected given its derivation within AF populations and its reflection of myocardial stress, the novel contribution of this study lies elsewhere. Specifically, we demonstrate that coronary-derived clinical and angiographic scores—originally unrelated to arrhythmia detection—show coherent, graded, and internally consistent associations with AF presence and phenotype in CCS. Importantly, the ABC-stroke score was not evaluated as a model for AF detection in the present study. Instead, it was incorporated as a biological comparator reflecting myocardial stress and injury, allowing exploration of whether biomarker-defined cardiovascular vulnerability converges with structural coronary complexity and traditional cardiovascular risk burden in CCS patients.

These observations support the concept that AF in CCS represents an integrated manifestation of systemic cardiovascular disease rather than an isolated electrical disturbance. This approach is supported by a clear pathophysiological rationale: AF is frequently progressive present, initially manifesting as an intermittent arrhythmia with silent periods of abnormal electrical activity that remain undetected in the absence of intensive rhythm monitoring. Large trials such as ASSERT and LOOP have demonstrated that up to 30% of patients at high vascular risk develop silent AF detectable only through continuous monitoring. Consequently, the development of a clinical algorithm based on parameters already collected in routine practice may represent an important strategy for screening and secondary prevention [21,22].

### 4.1. Atrial Fibrillation as an Expression of Systemic Cardiovascular Disease

Large cohort studies, including the Framingham Heart Study and ARIC, have demonstrated that hypertension, diabetes, dyslipidemia, systemic inflammation, and advancing age increase the risk of both coronary atherosclerosis and AF. Within this framework, AF may be considered the final result of a sequence of pathological processes—namely inflammatory activation, endothelial dysfunction, structural atrial remodeling, and ultimately loss of atrial electrical stability—underscoring the importance of early identification in determining long-term patient outcomes [14,23].

The findings of the present work align with this paradigm, demonstrating a graded progression of both clinical and anatomical scores with the transition from SR to paroxysmal, persistent, and permanent AF. Comparative analysis of risk scores between patients in SR and those with different AF phenotypes revealed a consistent and progressive pattern. Across all metrics, the lowest values were observed in SR, increased in paroxysmal AF, reached a peak in persistent AF, and remained elevated in permanent AF.

This progressive behavior was evident across multiple scores:

- SCORE2-OP, with significant increases from 18.9 in SR to 27.8 in paroxysmal AF (*p* = 0.004);
- Framingham risk score, where the mean increase from SR to paroxysmal AF exceeded 65% (*p* = 0.003);
- ASCVD, in which paroxysmal AF nearly doubled estimated risk (35.5 vs. 19.5, *p* < 0.001);
- SYNTAX PCI and SYNTAX CABG, which demonstrated progressively higher values across AF phenotypes, reflecting increasing coronary anatomical complexity in parallel with rhythm burden (*p* = 0.007 and *p* = 0.001, respectively);
- ABC score, which—serving here as a biomarker-based reference layer—exhibited the expected gradient across rhythm categories (*p* < 0.001), consistent with its reflection of myocardial stress and biological proximity to AF-related processes.

These results support the hypothesis that AF does not occur randomly during the course of coronary artery disease but rather represents an evolutionary threshold in patients with a high metabolic, vascular, and inflammatory burden. Moreover, this consistent pattern is concordant with observations from EHRA and ESC registry analyses, which identify AF as a phenotypic marker of structural cardiovascular aging [24,25].

An additional finding of particular interest—frequently highlighted in contemporary literature—is that paroxysmal AF appears to concentrate the highest systemic risk, as assessed by scores such as SCORE2/OP, Framingham, and ASCVD. In the present study, patients with paroxysmal AF exhibited the highest clinical risk scores in most analyses, in some cases exceeding those observed in persistent or permanent AF. This phenomenon has been confirmed in post hoc analyses of major trials such as AFFIRM and ATHENA, both of which reported a continuous increase in inflammatory markers and progressive deterioration of the electrical substrate during early and intermittent AF stages, before the arrhythmia becomes permanent. Paroxysmal AF may therefore represent a critical window during which preventive intervention is most effective, further justifying the use of clinical risk scores as tools for early detection [26,27].

### 4.2. Clinical and Angiographic Risk Scores—Repurposed Tools with Unexpected Utility

The ABC-stroke score and CCS intersect through shared pathophysiological mechanisms and overlapping cardiovascular vulnerability markers, particularly in patients with AF. By incorporating high-sensitivity cardiac troponin and NT-proBNP, the ABC framework captures ongoing myocardial injury and wall stress, thereby reflecting an active biological dimension of cardiovascular risk. Unlike traditional clinical scores based exclusively on cumulative historical exposure to risk factors, the ABC model integrates dynamic markers of myocardial stress. This characteristic explains its established role in thromboembolic risk stratification among AF populations, including those with coexisting CCS. In the present study, both the ABC-stroke score and the SYNTAX CABG score remained independently associated with AF in multivariable analysis. The strong association observed for the ABC score (OR = 35.16; AUC = 0.908) is biologically coherent with its derivation within AF cohorts and its proximity to myocardial stress pathways implicated in atrial remodeling. Importantly, the independent contribution of SYNTAX CABG underscores the relevance of structural coronary complexity beyond biomarker-defined vulnerability, suggesting that anatomical disease burden and biological stress operate in parallel [2,10].

The observed performance of the ABC framework is consistent with data from large registries such as GARFIELD-AF and ENGAGE AF-TIMI, where biomarker-integrated models demonstrated superior discrimination for adverse cardiovascular outcomes and reflected systemic frailty within AF populations. In this context, the strong association between ABC-stroke and AF status in CCS supports the concept of biological convergence rather than isolated predictive overlap [28,29].

Similarly, SYNTAX scores showed robust associations with AF (AUC 0.745–0.760), with particularly strong performance in persistent and permanent AF phenotypes. These findings align with prior reports indicating that greater coronary complexity is associated with AF development. Studies evaluating patients undergoing coronary artery bypass grafting have identified SYNTAX score as an independent predictor of postoperative AF, emphasizing the contribution of diffuse atherosclerotic burden to atrial arrhythmogenic substrate formation. Large observational data have further demonstrated that higher SYNTAX values are independently associated with AF occurrence following PCI, suggesting that complex coronary anatomy may coexist with and potentially promote atrial remodeling even in stable ischemic settings [9,11].

Beyond biomarker-defined vulnerability and anatomical complexity, global clinical risk scores such as Framingham, ASCVD, SCORE2 and SCORE2-OP also demonstrated structured associations with AF phenotype. These models, although originally developed for prediction of atherosclerotic cardiovascular events, integrate core determinants of atrial remodeling, including age, hypertension, diabetes, dyslipidemia, and smoking status. The graded increase observed particularly in paroxysmal AF suggests that cumulative cardiometabolic exposure may contribute to the early phases of atrial structural and electrical remodeling. Unlike the ABC framework, which captures active myocardial stress, and SYNTAX, which reflects anatomical disease complexity, clinical risk scores represent a cumulative and longitudinal dimension of cardiovascular burden. Their association with AF in CCS supports the concept that AF emerges at the intersection of metabolic risk, structural coronary disease, and biological myocardial stress. Thus, the alignment observed across these diverse scoring systems reinforces the hypothesis that AF in CCS is not an isolated arrhythmia but rather a systemic cardiovascular phenotype. Several large-scale studies in the literature further support the concept that integrated cardiovascular risk burden and structural factors contribute to the development of AF. A landmark Framingham Heart Study-derived risk score for new-onset AF identified traditional clinical risk factors—including age, hypertension, diabetes mellitus, and coronary heart disease—as strong independent predictors over a 10-year follow-up period, providing a validated framework for AF risk stratification in community populations and highlighting the overlap between coronary risk and arrhythmia risk profiles. Moreover, another study has also underscored the relevance of composite risk models in predicting cardiovascular events and arrhythmic outcomes. For example, in hypertensive patients undergoing catheter ablation, a SCORE2/SCORE2-OP CVD risk ≥ 10% was independently associated with AF recurrence and adverse cardiovascular outcomes, suggesting that global atherosclerotic risk estimates may track with arrhythmia propensity. Furthermore, broader epidemiological reviews emphasize that modifiable cardiovascular risk factors—such as hypertension, obesity, and diabetes—are mechanistically linked to atrial remodeling, structural changes, and increased AF incidence, reinforcing the rationale for integrating global risk assessment into AF prediction strategies. In this context, our findings align with and extend existing evidence by demonstrating that clinical and angiographic scores used in CCS not only reflect coronary disease severity but also associate significantly with AF presence and phenotype. This supports the idea that AF in the setting of chronic coronary disease may reflect an integrated cardiovascular risk profile rather than a discrete electrical abnormality [30,31,32].

In contrast, the Gensini score did not demonstrate a significant association with AF presence or subtype, highlighting important conceptual differences between anatomical scoring systems. While Gensini quantifies the percentage severity of focal coronary stenoses, it does not capture lesion multiplicity, diffuse involvement, vascular network complexity, or overall anatomical heterogeneity. AF, however, is increasingly recognized as a manifestation of systemic cardiovascular remodeling characterized by inflammation, endothelial dysfunction, oxidative stress, and microvascular impairment. In this context, focal stenotic severity alone may insufficiently reflect the structural and hemodynamic milieu contributing to atrial remodeling. The weak correlations observed between Gensini and AF (rho < 0.25) likely reflect this pathophysiological limitation. Taken together, these findings reinforce the distinction between focal stenosis severity and global coronary complexity. While Gensini remains valuable for quantifying isolated anatomical obstruction, SYNTAX-based assessments—by integrating lesion distribution, multiplicity, and structural complexity—appear more closely aligned with the diffuse cardiovascular substrate underlying AF. The graded increase in SYNTAX scores across AF phenotypes, coupled with their independent association in multivariable models, supports the concept that AF in CCS reflects cumulative structural and biological cardiovascular burden rather than an isolated electrical disturbance [7].

### 4.3. Correlations Between Scores—Pathophysiological Convergence and Structural Limitations

Spearman correlation analysis performed in the present study provides additional insight into how different clinical, metabolic, and anatomical scores reflect the global cardiovascular disease burden in the context of AF. The results outline a coherent landscape of relationships between risk factors, coronary complexity, and arrhythmia, highlighting both convergence among certain scores and limitations among others. Robust correlations were observed between global cardiovascular risk scores—SCORE2-OP, ASCVD, and Framingham—all of which are based on cumulative traditional risk factors. Moderate to strong relationships (rho 0.34–0.68, *p* < 0.001) are unsurprising, given that these tools rely on similar pillars: age, hypertension, diabetes, dyslipidemia, and smoking—key contributors to both coronary atherosclerosis and the electrical substrate predisposing to AF. As emphasized by the 2021 ESC Guidelines on Cardiovascular Prevention, risk factor-based scores essentially quantify “cumulative etiological burden,” reflecting chronic exposure to disease-driving mechanisms [33].

Importantly, these risk factor-based scores also demonstrated significant and consistent correlations with anatomical SYNTAX PCI and CABG scores (rho 0.32–0.63, all *p* < 0.001). This convergence between clinical risk and morphological expression suggests that patients with AF are not only metabolically or inflammatorily predisposed but also exhibit angiographically detectable structural cardiac changes. The significant correlation with SYNTAX—but not necessarily with Gensini—indicates that AF is associated with diffuse, segmental, and complex coronary disease rather than isolated focal stenosis [34].

Overall, Spearman analysis fulfills a key role by confirming that: SCORE2-OP, ASCVD, and Framingham capture predisposition; SYNTAX PCI/CABG capture disease expression and Gensini, limited to focal severity, loses sensitivity in diffuse disease—precisely the scenario typical of AF. This supports the conceptual validity of the thesis objective: AF is not an isolated phenomenon, but an integrated manifestation of an atherosclerotic and inflammatory continuum that can be anticipated through pathogenetically aligned scoring systems.

### 4.4. Clinical Utility and Practical Implications

The data obtained in the present study suggest that the analyzed scores may be used not only to describe the profile of patients with AF, but also to identify those in whom AF is likely present yet undetected. Clinical and anatomical scores may therefore shift AF management from a reactive diagnostic-therapeutic approach toward a proactive strategy. A major clinical message emerging from this analysis is that as risk scores increase, rhythm monitoring should be intensified, even in the absence of documented AF on standard electrocardiography. This conclusion aligns with evidence from studies such as ASSERT, LOOP, and mHealth-AF, which have demonstrated that the presence of advanced vascular or structural substrate dramatically increases the likelihood that AF is already present in a subclinical form [21,27,35].

Exploratory threshold analysis identified values potentially associated with increased arrhythmic vulnerability within this cohort, including an ABC-stroke score ≥ 0.60, SYNTAX PCI or CABG scores > 30, and ASCVD or Framingham scores within the upper quartile of distribution. Although these cut-off values are not intended to represent formal clinical decision thresholds, they may provide preliminary signals for identifying CCS patients with heightened susceptibility to AF. Such findings should be interpreted as hypothesis-generating and warrant validation in larger prospective cohorts before clinical implementation. Future studies may determine whether these parameters could assist in refining rhythm surveillance strategies in high-risk CCS populations.

This paradigm shift implies risk-stratified monitoring strategies that may include periodic resting ECGs, 48–72 h Holter monitoring at 6–12-month intervals, and—in very high-risk patients, particularly those aged ≥75 years with multivessel coronary disease, elevated SYNTAX scores, or persistent clinical suspicion—implantable loop recorders. The clinical consequences of early AF identification are substantial, as prompt detection enables preventive initiation of anticoagulation in eligible patients, stroke prevention—given that 20–30% of cryptogenic strokes are attributable to undiagnosed AF, optimization of blood pressure control and other risk factors and early implementation of rhythm or rate control strategies to limit atrial remodeling and prevent tachycardiomyopathy. These benefits are supported by recent evidence, including the EAST-AFNET 4 trial, which demonstrated that early AF treatment reduces cardiovascular hospitalizations and the risk of heart failure or stroke, underscoring the value of active screening in high-risk populations [36].

### 4.5. Strengths and Study Limitations

Several limitations should be acknowledged. This was a single-center study, which may limit generalizability, as cardiovascular risk profiles and referral patterns may vary across populations. The relatively modest sample size (*n* = 131) restricts statistical power, particularly in subgroup analyses by AF phenotype, and may limit detection of weaker associations so it requires that the findings be interpreted as exploratory.

The cross-sectional design precludes assessment of temporal relationships between risk score elevation and AF development; therefore, causal inferences cannot be established. Moreover, the evaluated clinical and anatomical scores were not originally developed to predict AF in patients with CCS, and their application in this context should be considered exploratory and hypothesis-generating. Although ROC analysis was performed to explore the discriminative behavior of the evaluated scores, the study was not designed to develop a formal predictive model for AF. Consequently, advanced validation techniques such as net reclassification improvement or decision curve analysis were not applied, and the findings should be interpreted as exploratory.

A potential selection bias related to referral for invasive coronary angiography must also be considered, as the study population may represent a higher-risk subset of CCS patients. Then, although rhythm assessment included standard ECG and 24 h Holter monitoring when clinically indicated, systematic prolonged rhythm monitoring was not performed in all patients. Therefore, the possibility of undetected paroxysmal AF cannot be completely excluded.

Several additional variables known to influence AF risk, including left atrial size, left ventricular diastolic dysfunction, obesity, sleep apnea, and inflammatory biomarkers, were not included in the present analysis because their evaluation was beyond the specific aim of this study. The association of these parameters with AF has already been well established in previous research. However, the absence of adjustment for these potential confounders should be acknowledged when interpreting the observed associations and represents a limitation of the current analysis.

Finally, the absence of longitudinal follow-up precludes evaluation of incident AF, AF progression, or long-term cardiovascular outcomes. Prospective, multicenter studies are required to validate these findings and clarify their clinical implications.

## 5. Conclusions

In conclusion, in this proof-of-concept cross-sectional study, clinical and angiographic risk scores routinely used in CCS demonstrated coherent and graded associations with the presence and phenotype of AF. The behavior of the biomarker-based ABC reference model, together with the consistent alignment between cardiovascular risk burden and coronary anatomical complexity, supports a layered framework in which AF represents an integrated expression of systemic cardiovascular disease rather than an isolated electrical disorder. These findings should be considered hypothesis-generating and may provide a conceptual rationale for risk-enriched rhythm surveillance strategies in CCS populations. Confirmation in larger prospective and longitudinal studies is warranted to determine their predictive and clinical implications.

## Figures and Tables

**Table 1:** Risk scores used in CCS and their calculation methods [6,14,15,16].

Risk Score	Score Type	Components and Calculation Method
Gensini	Anatomical (angiographic)	Each coronary lesion is assigned a score according to the degree of stenosis: 25% (1 point), 50% (2 points), 75% (4 points), 90% (8 points), 99% (16 points), total occlusion (32 points); the score is subsequently weighted based on lesion location (e.g., left main coronary artery, left anterior descending artery); the final score represents the sum of all lesion scores
SYNTAX	Anatomical (lesion complexity)	Assesses each coronary lesion ≥ 50% in vessels ≥ 1.5 mm; incorporates lesion location, bifurcations/trifurcations, chronic total occlusions, severe calcification, vessel tortuosity, thrombus presence, and diffuse disease; the final score reflects the overall complexity of coronary anatomy (https://syntaxscore.org/calculator/syntaxscore/frameset.htm accessed on 15 May 2024)
Framingham	Clinical (global cardiovascular risk and primary prevention)	Age; sex; total cholesterol; HDL cholesterol; systolic blood pressure; antihypertensive treatment; smoking; diabetes mellitus; estimates 10-year risk of coronary events
ASCVD	Clinical (atherosclerotic risk and primary prevention)	Age; sex; race; total cholesterol; HDL cholesterol; systolic blood pressure; antihypertensive treatment; diabetes mellitus; smoking; estimates 10-year risk of myocardial infarction and stroke
SCORE2	Clinical (primary and secondary prevention in Europe)	Age (40–69 years); sex; smoking; systolic blood pressure; non-HDL cholesterol; estimates 10-year risk of fatal and non-fatal cardiovascular events, regionally calibrated
SCORE2-OP	Clinical (older populations) in Europe	Age ≥70 years; sex; smoking; systolic blood pressure; non-HDL cholesterol; adaptation of SCORE2 for older individuals, estimating 10-year cardiovascular risk
ABC-stroke risk score	Clinical	Age, prior stroke or transient ischemic attack, high-sensitivity cardiac troponin, NT-proBNP, from Uppsala Clinical Research Center (https://www.ucr.uu.se/en/services/abc-risk-calculators—accessed on 21 May 2024)

CCS: chronic coronary syndrome; HDL: high-density lipoproteins; ASCVD: Atherosclerotic Cardiovascular Disease score; SCORE2: Systematic Coronary Risk Estimation 2; SCORE2-OP: Systematic Coronary Risk Estimation 2—older persons; NT-proBNP: N-terminal pro–B-type natriuretic peptide.

**Table 2:** Distribution of patients with AF according to CCS severity.

		CCS				Total		Pearson Chi-Squared Test
		N-CCS		S-CCS				
		*n*	%	*n*	%	*n*	%	
Rythm	SR	25	37.9%	35	53.8%	60	45.8%	Chi2 = 9.267
	PAROX	8	12.1%	14	21.5%	22	16.8%	*p* = 0.026 *
	PERS	9	13.6%	5	7.7%	14	10.7%	
	PERM	24	36.4%	11	16.9%	35	26.7%	
Total		66	100.0%	65	100.0%	131	100.0%	

* *p* < 0.05; CCS: chronic coronary syndrome; N-SCC: non-significant CCS; S-SCC: significant CCS; AF: atrial fibrillation; SR: sinus rhythm; PAROX: paroxysmal atrial fibrillation; PERS: persistent atrial fibrillation; PERM: permanent atrial fibrillation.

**Table 3:** Comparison of clinical risk scores in CCS according to AF type.

Parameter	Group	*n*	Mean ± SD	Median (IQR: 25–75)	*p* Value
Gensini score	SR	60	30.24 ± 38.64	16.50 (0.04–200.00)	0.043 *
	PAROX	22	30.86 ± 39.82	10.50 (2.50–154.00)	
	PERS	14	25.00 ± 32.68	8.00 (1.00–94.00)	
	PERM	35	15.29 ± 22.14	5.00 (1.00–100.00)	
	All types of AF	71	22.03 ± 31.01	8.00 (1.00–154.00)	0.052 ^+^ Post hoc: PAROX *versus* PERM *p* = 0.038 *; SR *versus* PERM *p* = 0.007 **
ABC score	SR	60	0.45 ± 0.31	0.36 (0.15–2.36)	<0.001 **
	PAROX	22	0.86 ± 0.53	0.76 (0.39–2.74)	
	PERS	14	0.90 ± 0.44	0.79 (0.49–2.07)	
	PERM	35	1.13 ± 0.63	0.88 (0.40–3.49)	
	All types of AF	71	1.00 ± 0.57	0.85 (0.39–3.49)	<0.001 ** Post hoc: *p* = 0.000 ** for SR *versus* all types of AF
Score 2/OP	SR	60	18.89 ± 10.42	16.50 (2.60–48.00)	0.004 **
	PAROX	22	27.86 ± 8.88	29.00 (15.00–47.00)	
	PERS	14	24.90 ± 14.86	21.00 (7.70–55.00)	
	PERM	35	21.08 ± 10.18	18.00 (3.10–41.00)	
	All types of AF	71	23.93 ± 11.14	21.00 (3.10–55.00)	0.006 * Post hoc: SR *versus* PAROX *p* = 0.002 *
Framingham score	SR	60	11.30 ± 7.60	10.15 (0.50–35.00)	0.003 **
	PAROX	22	18.65 ± 6.99	20.35 (5.10–29.10)	
	PERS	14	14.73 ± 10.34	12.55 (4.20–44.50)	
	PERM	35	13.61 ± 8.62	13.70 (1.20–31.30)	
	All types of AF	71	15.39 ± 8.70	15.10 (1.20–44.50)	0.007 ** Post hoc: SR *versus* PAROX *p* = 0.001 **
ASCVD score	SR	60	19.54 ± 13.23	18.05 (1.50–59.50)	<0.001 **
	PAROX	22	35.55 ± 14.10	32.40 (15.10–60.80)	
	PERS	14	21.62 ± 9.77	19.90 (4.80–39.70)	
	PERM	35	20.92 ± 12.45	18.50 (1.10–63.90)	
	All types of AF	71	25.59 ± 14.07	21.30 (1.10–63.90)	0.010 * Post hoc: SR *versus* PAROX *p* = 0.000 **; PERM *versus* PAROX *p* = 0.002 *
SYNTAX PCI	SR	35	29.50 ± 8.82	26.80 (16.0–51.7)	0.007 **
	PAROX	14	35.90 ± 8.62	31.85 (25.5–51.3)	
	PERS	5	43.44 ± 13.28	45.60 (22.8–57.6)	
	PERM	11	38.81 ± 12.97	34.60 (20.3–67.4)	
	All types of AF	71	38.22 ± 11.09	36.30 (20.3–67.4)	<0.001 **
SYNTAX CABG	SR	35	26.35 ± 9.04	26.30 (7.8–47.3)	0.001 **
	PAROX	14	34.90 ± 9.41	35.00 (21.6–54.2)	
	PERS	5	41.04 ± 11.44	39.10 (24.3–54.6)	
	PERM	11	34.31 ± 9.94	30.10 (23.5–53.6)	
	All types of AF	71	35.71 ± 9.89	35.00 (21.6–54.6)	<0.001 ** Post hoc: SR *versus* PAROX *p* = 0.029 *; SR *versus* PERS *p* = 0.010 *

* Statistically significant difference (*p* < 0.05); ** statistically highly significant difference (*p* < 0.01); ^+^ value close to statistical significance; SR: sinus rhythm; PAROX: paroxysmal atrial fibrillation; PERS: persistent atrial fibrillation; PERM: permanent atrial fibrillation; AF: atrial fibrillation.

**Table 4:** Multivariate analysis: risk enrichment for AF detection.

	B	S.E.	Wald	df	Sig.	Exp(B) = OR	95% CI for Exp (B)	
							Lower	Upper
ABC score	3.560	1.154	9.512	1	0.002 **	35.160	3.660	337.734
SYNTAX CABG	0.077	0.038	4.208	1	0.040 *	1.080	1.003	1.163
Constant	−1.069	0.772	1.918	1	0.166	0.343		

* Statistically significant difference (*p* < 0.05); ** statistically highly significant difference (*p* < 0.01).

**Table 5:** ROC analysis results for parameters showing statistically significant variations in patients with atrial fibrillation.

	AUC	*p*	95% CI		Cut-off Value	Sensibility	Specificity
			Lower	Upper			
Gensini (-) score	0.598	0.053	0.501	0.696	10.50	0.592	0.667
ABC	0.908	0.000 **	0.854	0.961	0.615%	0.831	0.883
SCORE 2/OP	0.639	0.006 **	0.543	0.734	12.50%	0.887	0.383
FRAMINGHAM	0.638	0.007 **	0.543	0.732	15.05%	0.507	0.733
ASCVD	0.631	0.010 *	0.535	0.727	16.55%	0.761	0.483
SYNTAX PCI	0.745	0.001 **	0.625	0.865	28.95	0.833	0.629
SYNTAX CABG	0.760	0.000 **	0.644	0.875	29.95	0.700	0.743

* Statistically significant difference (*p* < 0.05); ** statistically highly significant difference (*p* < 0.01).

**Table 6:** Pearson correlations between clinical scores, cardiovascular risk scores, and angiographic indicators.

Confidence Intervals for the Spearman Rho Coefficient				
	Spearman’s Rho	*p*	95% CI	
			Lower	Upper
Gensini—ABC	−0.021	0.810	−0.197	0.156
Gensini—SCORE 2/OP	0.104	0.237	−0.074	0.276
Gensini—FRAMINGHAM	0.201	0.021 *	0.025	0.365
Gensini—ASCVD	0.298	<0.001 **	0.128	0.451
Gensini—SYNTAX PCI	0.246	0.049 *	−0.006	0.468
Gensini—SYNTAX CABG	0.225	0.072	−0.027	0.450
ABC—SCORE 2/OP	0.260	0.003 **	0.088	0.418
ABC—FRAMINGHAM	0.152	0.083	−0.025	0.320
ABC—ASCVD	0.196	0.025 *	0.020	0.360
ABC—SYNTAX PCI	0.559	<0.001 **	0.358	0.710
ABC—SYNTAX CABG	0.491	<0.001 **	0.273	0.660
SCORE 2/OP—FRAMINGHAM	0.344	<0.001 **	0.178	0.491
SCORE 2/OP—ASCVD	0.681	<0.001 **	0.574	0.766
SCORE 2/OP—SYNTAX PCI	0.593	<0.001 **	0.402	0.735
SCORE 2/OP—SYNTAX CABG	0.627	<0.001 **	0.447	0.759
FRAMINGHAM—ASCVD	0.656	<0.001 **	0.542	0.746
FRAMINGHAM—SYNTAX PCI	0.058	0.648	−0.196	0.304
FRAMINGHAM—SYNTAX CABG	0.422	<0.001 **	0.191	0.608
ASCVD—SYNTAX PCI	0.321	0.009 **	0.077	0.530
ASCVD—SYNTAX CABG	0.572	<0.001 **	0.375	0.719
SYNTAX PCI—SYNTAX CABG	0.663	<0.001 **	0.495	0.784

* Statistically significant difference (*p* < 0.05); ** statistically highly significant difference (*p* < 0.01).

## Data Availability

The original contributions presented in this study are included in the article. Further inquiries can be directed to the corresponding author.
